# Improved efficacy of naproxen-loaded NLC for temporomandibular joint administration

**DOI:** 10.1038/s41598-019-47486-w

**Published:** 2019-08-01

**Authors:** Viviane A. Guilherme, Lígia N. M. Ribeiro, Ana C. S. Alcântara, Simone R. Castro, Gustavo H. Rodrigues da Silva, Camila Gonçalves da Silva, Márcia C. Breitkreitz, Juliana Clemente-Napimoga, Cristina G. Macedo, Henrique B. Abdalla, Ricardo Bonfante, Cintia M. S. Cereda, Eneida de Paula

**Affiliations:** 1 Department of Biochemistry and Tissue Biology, Institute of Biology, University of Campinas (UNICAMP), Campinas, São Paulo Brazil; 2 Department of Chemistry, Federal University of Maranhão – UFMA, São Luis, Maranhão Brazil; 3 Department of Analytical Chemistry, Institute of Chemistry, UNICAMP, Campinas, São Paulo Brazil; 4 Department of Physiological Science, Piracicaba Dental School, University of Campinas, Piracicaba, São Paulo Brazil; 5 Faculdade São Leopoldo Mandic, Instituto de Pesquisas São Leopoldo Mandic, Campinas, São Paulo Brazil

**Keywords:** Nanobiotechnology, Drug delivery

## Abstract

Inflammatory conditions of the temporomandibular joint (TMJ) and peripheral tissues affect many people around the world and are commonly treated with non-steroidal anti-inflammatory drugs (NSAIDs). However, in order to get desirable results, treatments with NSAIDs may take weeks, causing undesirable side effects and requiring repeated administration. In this sense, this work describes the development of an optimized nanostructured lipid carrier (NLC) formulation for intra-articular administration of naproxen (NPX). An experimental design (2^3^) selected the best formulation in terms of its physicochemical and structural properties, elucidated by different methods (DLS, NTA, TEM, DSC, and ATR-FTIR). The chosen formulation (NLC-NPX) was tested on acute inflammatory TMJ nociception, in a rat model. The optimized excipients composition provided higher NPX encapsulation efficiency (99.8%) and the nanoparticles were found stable during 1 year of storage at 25 °C. *In vivo* results demonstrated that the sustained delivery of NPX directly in the TMJ significantly reduced leukocytes migration and levels of pro-inflammatory cytokines (IL-1β and TNF-α), for more than a week. These results point out the NLC-NPX formulation as a promising candidate for the safe treatment of inflammatory pain conditions of TMJ or other joints.

## Introduction

Inflammation in the temporomandibular joint (TMJ) affects around 10% of the world population^1,2^. The inflammatory process of the TMJ results in the release of several pro-inflammatory cytokines, prostanoids, and sympathetic amines that contribute to joint remodeling, cartilage degradation, and maintenance of a painful condition^3^. Nonsteroidal anti-inflammatory drugs (NSAIDs), such as naproxen (NPX), are commonly prescribed for the treatment of patients with temporomandibular disorders such as arthritis/arthrosis to restore function, limiting the disease progression^4^. NPX acts by inhibiting cyclooxygenase-1 and 2 (COX 1 and 2) that are responsible for PGE2 production when stimulated by inflammatory mediators, such as the tumor necrosis factor and interleukins^5^.

The intra-articular injection is the most efficient route for drug administration in TMJ. However, this route shows several disadvantages, including high frequency of drug administration, which causes pain and decreases the patient compliance^6^. In this sense, the use of drug delivery systems (DDS) seems to be a versatile approach to overcome such limitations in the traditional treatment of this inflamed region.

Nanostructured lipid carriers (NLC) is a promising DDS able to prolong the delivery, improve the stability, and decrease the systemic toxicity of liposoluble drugs^7,8^. These innovative nanoparticles, formed by the combination of at least one solid and one liquid lipid (at body temperature) plus a surfactant, can be used in several areas, such as in pharmacy and biotechnology^9,10^. Encapsulation of drugs with anti-inflammatory properties into lipid nanoparticles has been successfully reported for different NSAIDs^11,12^ and thymol^13^, which were able to decrease the administration frequency, improving the therapeutic efficacy. Moreover, the biocompatible nature of the lipids that compose the NLC may contribute to decrease drug local toxicity, and subsequent inflammation inherent to injection at TMJ^14^.

This work reports the development and characterization of an optimized NLC-NPX formulation designed for administration into TMJ. The system was stable for one year (25 °C), with excellent physicochemical properties. The sustained release profile provided by the NLC-NPX formulation enhanced the efficacy and bioavailability of naproxen, resulting in prolonged (one week) peripheral anti-inflammatory effect on TMJ.

## Results

### Experimental design

The first step in the development of NLC is the lipid matrix selection. In a preliminary test, the mixture of cetyl palmitate (CP) and capric/caprylic triglycerides (CCT) displayed high dissolution capacity for NPX^15^. Pluronic^®^ F68 (P68) was the chosen surfactant, being able to stabilize the nanoparticles by steric repulsion^16^. The concentration range of the excipients used in the NLC preparation can be seen in Table S1.

**Figure 1:**
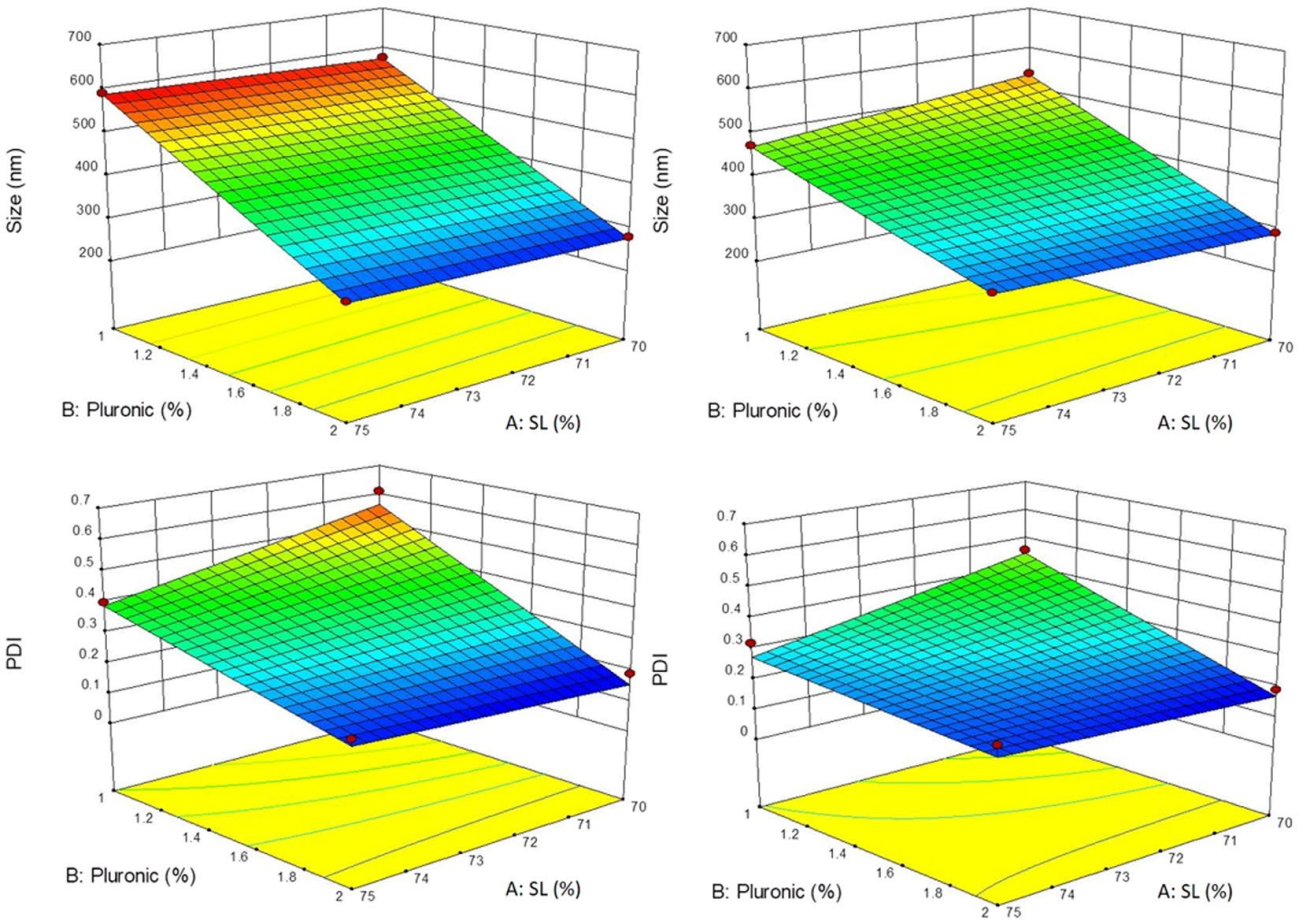
Surface plots for the responses: average diameter (Size - top) and polydispersity index (PDI - bottom) of NLC formulations without naproxen (left) and with 3% naproxen (right). P68 (%) = surfactant percentage; SL (%) = solid lipid percentage.

NLC formulations with particle sizes between 279–591 nm and variable polydispersity index (PDI = 0.19–0.61) (Table S2) were obtained. Table S3 reveals that the linear regression was significant (p < 0.05) for both responses, the lower p-values for the “size” response indicating that the experimental variables exert a stronger influence on the nanoparticles diameter than in PDI. Table S3 also shows that the lack of fit of linear models was not significant (p > 0.05). Table S4 discloses details on the influence of each variable on the “size” response; for instance: the increase in P68 and NPX concentrations caused a decrease in particle size (negative effect) and also the interaction between these two factors significantly affected the nanoparticles size. As for the PDI response (Table S5), only P68 exerted a significant influence, contributing to a monodisperse distribution at increasing concentrations. Figure 1 sums up all these results, trough surface plots for the NLC particle size and PDI, evidencing the effects of P68 and solid lipid percentage (SL %) on these responses.

In order to select formulations with suitable particle size (<300 nm) and polydispersity (<0.2), the desirability procedure was used (Fig. S1). The chosen formulation was composed of 20% total lipids (70/30 solid lipid/liquid lipid), 2% P68, and 3% naproxen (NLC-NPX), as highlighted in Table S2. The encapsulation efficiency (%EE) of naproxen in the optimized system was excellent (99.8 ± 0.2%) justifying its selection for further investigation.

### Physicochemical characterization of the optimized NLC-NPX formulation

Dynamic Light Scattering (DLS) and Nanoparticle Tracking Analysis (NTA) were used to characterize the optimized NLC-NPX formulation and its control, prepared without naproxen (NLC).

The selected optimized formulation presented particle size around 278.4 ± 5.2 nm for NLC and 289.5 ± 3.6 nm for NLC-NPX. In both cases, low PDI values (0.190 ± 0.007) were registered. The zeta potential (ZP) became more negative in the presence of NPX, with values around −11.5 ± 0.5 mV and −15.4 ± 2.9 mV, for NLC and NLC-NPX, respectively. These values can be seen and are discussed below, with other data related to the physicochemical stability of the formulations during storage.

Particle size was determined also by NTA (Table S6). For both formulations (with and without NPX) the average diameters measured by NTA were found smaller than those determined by DLS (formulations 3 and 7 - Table S2). Most interestingly, NTA also provided information regarding nanoparticle concentration (particle/mL). The number of nanoparticles increased from 3.0 × 10^13^ to 4.7 × 10^13^ particles/mL in the presence of NPX (Table S6). From this data and considering the molar concentration of the excipients, we could estimate the number of CP, CCT, P68 and NPX molecules in a single NLC^9,17^. The calculated composition (Table S6) revealed that NPX encapsulation into the nanoparticle was followed by a decrease in the number of excipient molecules (CP, CCT, P68) per NLC.

### Transmission electron microscopy

**Figure 2:**
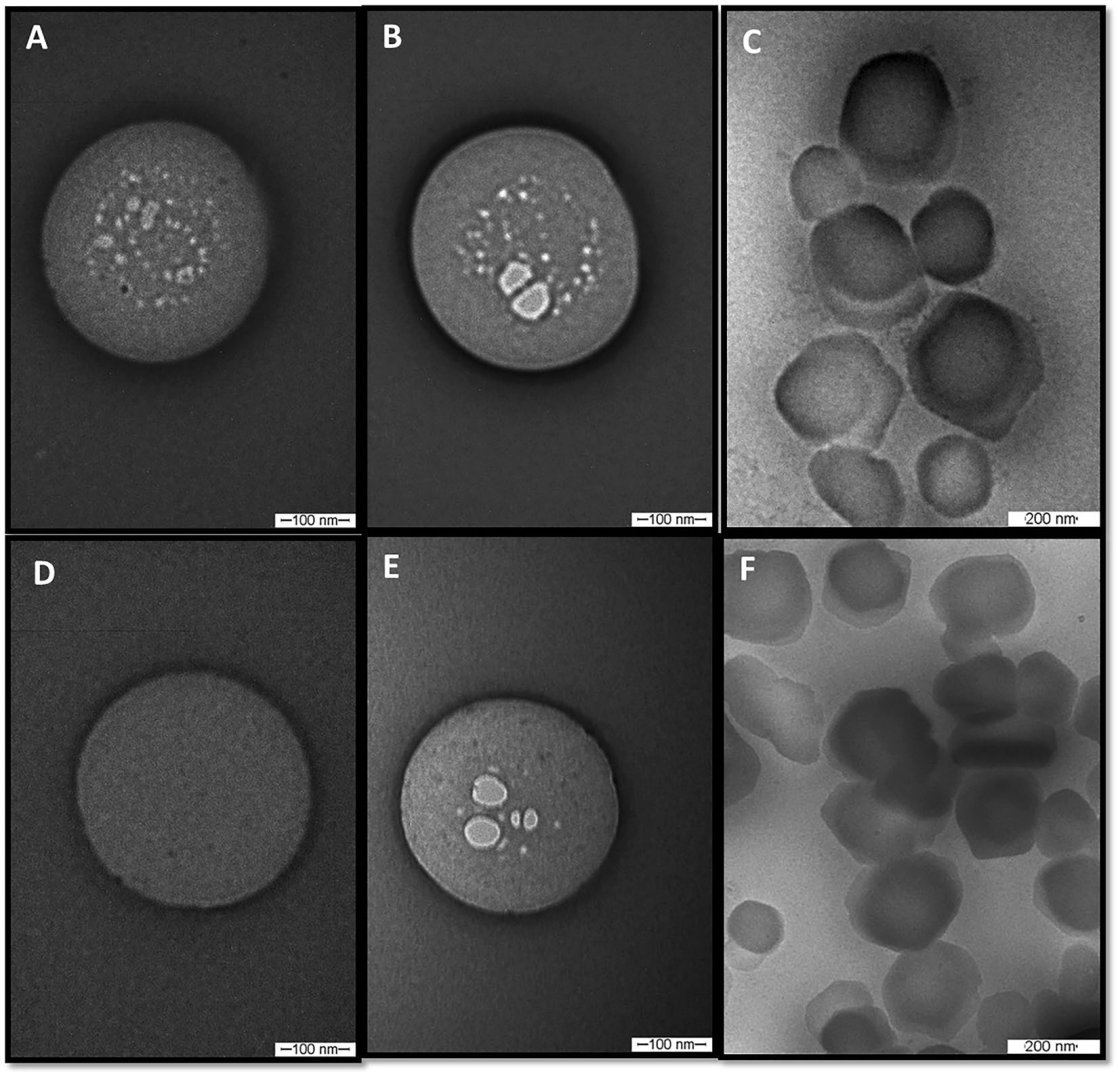
TEM images of nanoparticles after 5 days of preparation (top) and after 12 months of storage at room temperature (bottom). (**A,D**) = NLC; (**B,C,E,F**) = NLC-NPX. Magnifications: (**A,B,D,E**) = 100,000x; (**C,F**) = 60,000x.

TEM images disclosed the spherical morphology (Fig. 2) of the optimized NLC, and particle sizes in agreement with DLS and NTA data. Indeed, the micrographs in low magnification (Fig. 2C,F) corroborated the monodisperse distribution (low PDI values) measured by DLS. TEM images also show that NPX encapsulation (Fig. 2B,E) did not change the morphology/size of the nanoparticles (Fig. 2A,D). Finally, it is noticeably the morphological similarity between images obtained with freshly prepared samples (5 days - Fig. 2A–C) and those stored for one year (Fig. 2D–F).

### ATR-FTIR and DSC analyses

The ATR-FTIR and DSC analyses were performed in order to clarify the molecular arrangement of the optimized NLC-NPX formulation, emphasizing the drug-excipient interactions.

**Figure 3:**
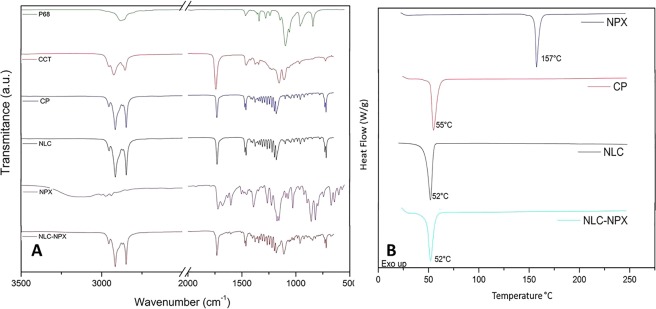
ATR-FTIR (**A**) and DSC (**B**) analyses of nanostructured lipid carriers (NLC) and naproxen-loaded NLC (NLC-NPX) formulations, and their excipients: cetyl palmitate (CP), capric/caprylic acid triglycerides (CCT), Pluronic^®^ F68 (P68), and naproxen (NPX).

Figure 3A shows the ATR-FTIR spectra of NPX, NLC, NLC-NPX, and their excipients. The spectrum of NPX showed typical bands at 3122, 1720, 1600, 1390, 1177, 854, and 673 cm^−1^, assigned to ʋC–H vibrations of the aliphatic chain, C=O, C=C, δCH_3_, δC–H in-plane, δC–H out-of-plane and δC–C (ring bending), respectively^18^. In the vibrational spectrum of P68 typical bands were detected: (i) of aliphatic chain ethers at 2878 and 1340 cm^−1^, related to the ʋO–CH_2_ and ʋO–C–O vibrations, respectively, (ii) the characteristic bands of symmetric deformation (δCH_2_) in 1469 cm^−1^, and (iii) the axial stretching of CO in 1094 cm^−1^ ^19^. As for the solid (CP) and liquid (CCT) lipids, typical aliphatic chains absorptions were observed, such as: (i) the bands in the region between 2915 and 2925 cm^−1^ and a set of bands in the region between 1462 and 1150 cm^−1^, assigned to CH_2_ vibrations, (ii) sharp bands centered at 1730 and 2850–2854 cm^−1^ and attributed to C=O and O–CH_2_ vibrations, as well as (iii) (CH_2_)_n_ vibrations, associated with the angular deformation (rocking) in the region between 720–728 cm^−1^.

It is worth mentioning that being CP the most abundant component of the nanoparticles, some of its typical bands could be detected in the spectra of NLC and NLC-NPX, such as those associated with CH_2_ (between 1457 and 1150 cm^−1^) and C=O (1729 cm^−1^) vibrations. In the NLC and NLC-NPX spectra, bands ascribed to CP, CCT and P68 were assigned. Due to the minor contribution of NPX (3%) to the formulation, only a few bands of low intensity (*e.g*. at 1600 cm^−1^, C=C)^20^ were observed in the NLC-NPX spectrum.

Figure 3B shows the thermodynamic properties of optimized formulations and their excipients. The thermogram of CP and NPX exhibited a single endothermic peak at 55 °C and 157 °C, respectively, corresponding to their melting points^10,21^. For the NLC and NLC-NPX formulations a single endothermic transition was detected at 52 °C, related to the melting point of cetyl palmitate^22^.

### Physicochemical stability during storage

**Figure 4:**
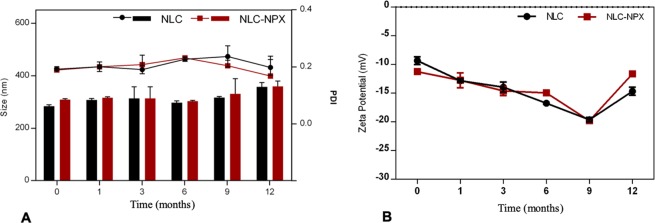
(**A**) Particle size (bars), PDI (lines), and (**B**) zeta potential values of the optimized NLC formulations, determined by DLS during 12 months of storage at 25 °C (n = 3). Data are displayed as mean ± S.D.

The long-term stability study of colloidal systems is of utmost importance to ensure the quality of the formulations^23^. The optimized (NLC and NLC-NPX) formulations were stored at room temperature for 12 months, and their particle size, PDI (Fig. 4A), and ZP values (Fig. 4B) were followed throughout this period.

The optimized formulations were found stable for 12 months, with no statistical differences (p > 0.05) in particle size - ranging from 284.2 to 358.8 nm (NLC) and 309.0 to 360.1 nm (NLC-NPX), at the initial and final times, respectively. The polydispersity of the particles size was also kept lower than 0.25 through the storage. Similarly, ZP values did not change significantly throughout the stability test, ranging from −11.5 (initial) to −11.6 mV (12 months) for NLC, and from −15.4 to 14.7 mV for NLC-NPX.

Additionally, the NPX encapsulation level into NLC measured after 12 months was 97.6 ± 0.1%, in good agreement with the initial data (99.8 ± 0.2%).

### *In vitro* naproxen release profile

**Figure 5:**
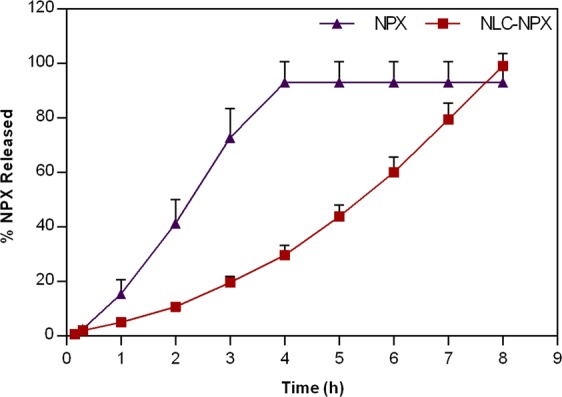
*In vitro* release profile of naproxen, measured at 37 °C. The NPX released was quantified by HPLC (n = 3). Data are displayed as mean ± S.D.

Figure 5 shows the release profile of NPX, either free or encapsulated into NLC. NPX was totally released from its hydroalcoholic solution after 4 h. On the other hand, a prolonged release (*ca*. 8 h) with no burst effect was observed in the NLC-NPX sample. The mathematical modeling of the NLC-NPX curve revealed a non-Fickian^24^ NPX release from the NLC formulation, according to the Korsmeyer-Peppas (r^2^ = 0.9909; k = 0.13, n = 0.96 – see Eq. 2) model.

### Cytotoxicity tests

**Figure 6:**
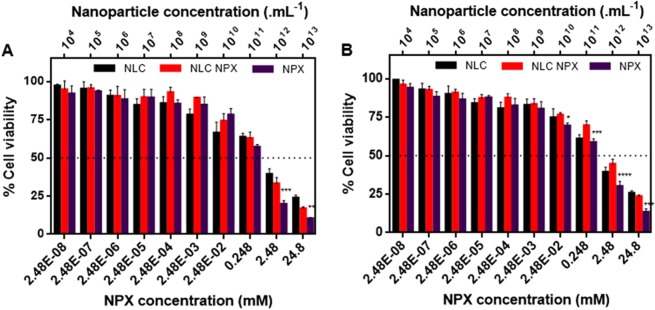
Cell viability tests – evaluated trough the MTT method - after treatment with naproxen (NPX), NLC-NPX or NLC (without NPX), for 24 h. (**A**) Balb/c 3T3 cells; (**B**) HaCat cells. One-way ANOVA Tukey post-test: * < 0.05, ** < 0.01, *** < 0.001, and **** < 0.0001.

Cell viability assays are widely employed to ascertain the safety of pharmaceutical formulations. Using two cell lines (3T3 and HaCat) we evaluated the percentage of viable cells after treatment for 24 h with increasing NPX (free and encapsulated) or NLC (without naproxen) concentrations (Fig. 6).

After 24 h of treatment, the drug concentrations for 50% viable cells (IC_50_ value) in 3T3 fibroblast cells (Fig. 6A) were 0.32 mM for NPX and 0.59 mM for NLC-NPX. In addition, the IC_50_ of HaCat keratinocyte cells (Fig. 6B) for NPX and NLC-NPX were 0.39 mM and 1.56 mM, respectively.

In order to evaluate the possible intrinsic toxicity of the nanoparticles, we prepared NLC samples containing the same lipid concentrations (0.0014 to 0.026% w:v) found in NLC-NPX samples. Figure 6A,B shows that a decrease in cell viability occurred at nanoparticle concentrations higher than 10^10^ NLC/mL. For 3T3 and HaCat cells, a 50% decrease in viable cells was assigned at 3.34.10^11^ and 2.98.10^11^ NLC/mL, respectively.

### Effect of NLC-NPX formulation on carrageenan-induced inflammation on TMJ

**Figure 7:**
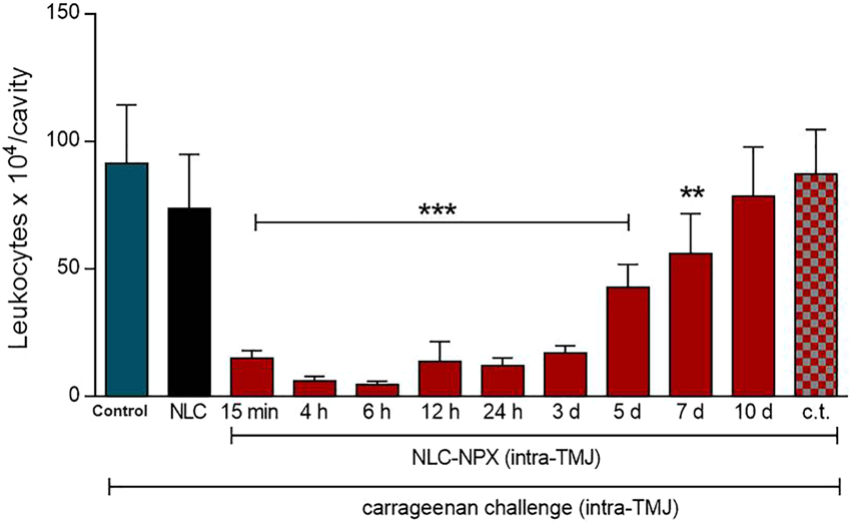
Effect of NLC-NPX on carrageenan-induced leukocyte migration in the TMJ of rats. Intra-TMJ injection of NLC-NPX significantly reduced the leukocyte migration induced by carrageenan. Data are expressed as mean ± SD. The symbols (***, **) means number of leukocytes significantly lower than those of animals treated with intra-TMJ injection of carrageenan. “c.t.” = contralateral treatment. The ANOVA and Tukey *post hoc* statistical analyses were performed (***p < 0.001, **p < 0.01).

The assessment of NLC-NPX ability to counter the inflammatory process was measured by the leukocyte infiltration of TMJ in rats. For that, the animals were pretreated with intra-articular injection (intra-TMJ, 20 uL) of NLC-NPX (0.6 mg) and challenged by intra-TMJ of carrageenan after 15 min, 4 h, 6 h, 12 h, 24 h, 3 d, 5 d, 7 d or 10 days. In comparison to control (carrageenan intra-TMJ injection), the pretreatment with NLC-NPX significantly reduced the carrageenan-induced leukocytes migration in the TMJ up to 7 days (Fig. 7). However, this anti-inflammatory property was abrogated on day 10 (Fig. 7, p > 0.05).

To further investigate if NLC-NPX action was local (not through systemic routes), another set of animals was pretreated with contralateral intra-TMJ injection of NLC-NPX. Nevertheless, the contralateral pretreatment was unable to blunt the leukocyte migration induced by carrageenan (Fig. 7, p > 0.05).

The protein level (low levels specifically) of pro-inflammatory cytokines are suitable markers of immune response suppression^25–27^, and NSAIDs have been shown to reduce such levels^28^. Therefore, to better elucidate the mechanism involved in the anti-inflammatory effect of NLC-NPX, we quantified the protein levels of two major pro-inflammatory cytokines, TNF-α and IL-1β in the periarticular TMJ tissue of rats.

**Figure 8:**
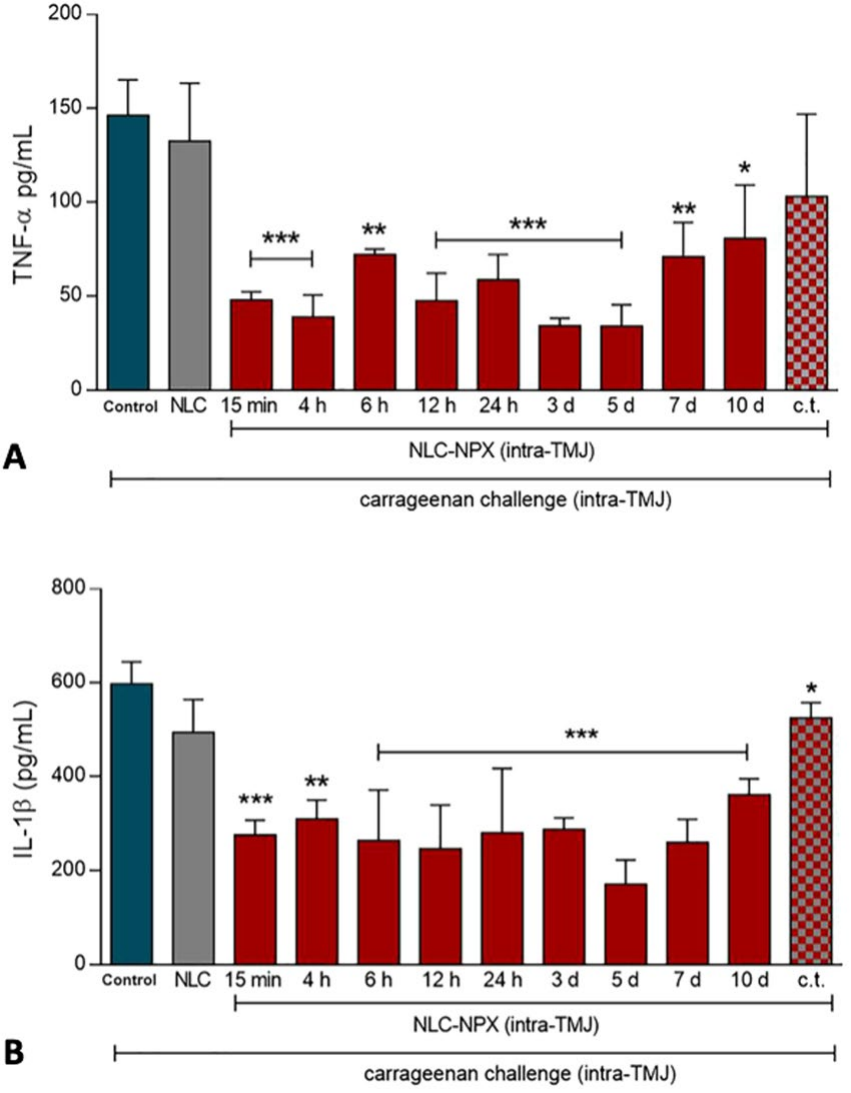
Effect of NLC-NPX on carrageenan-elicited protein level of inflammatory cytokines in the periarticular TMJ tissue of rats. Intra-TMJ injection of NLC-NPX significantly decreased the protein level of TNF-α (**A**) and IL-1β (**B**) in the periarticular tissue of rats. Data are expressed as mean ± SD. The symbols (***), (**) or (*) mean number of cytokines significantly lower than that of control animals, treated just with intra-TMJ injection of carrageenan (p < 0.001***, p < 0.01** or p < 0.05*: ANOVA, Tukey’s test). “c.t.” = contralateral treatment.

We have demonstrated that an intra-TMJ injection (20 μL) of NLC-NPX (0.6 mg) significantly reduced the carrageenan-elicited levels of TNF-α (Fig. 8A) and IL-1β (Fig. 8B). Moreover, the low levels of pro-inflammatory cytokines was sustained for 10 days (p < 0.05).

In addition, corroborating the previous findings on leukocyte migration, the NLC-NPX formulation elicited no systemic effect (Fig. 8A,B), since its administration into the contralateral TMJ did not reduce carrageenan-elicited protein level of pro-inflammatory cytokines (p > 0.05).

## Discussion

Pharmaceutical nanotechnology is a promising approach to solve several limitations of traditional drug therapy, such as side effects, poor bioavailability, and high frequency of NSAIDs administration. In the development of NLC formulations it is paramount to select the proper lipid matrix, considering its biocompatibility and miscibility with the drug of interest.

In this sense, experimental design has been widely employed in pharmaceutical development, as recommended by the FDA (United States Food and Drug Administration) and ICH (International Council for Harmonization of Technical Requirements for Pharmaceuticals for Human Use)^29,30^. The generated models for the significant factors are able to identify how they influence the critical quality attributes (CQAs) of the formulations, as well as to point out interactions among factors, allowing an effective optimization of the system. Desirable CQAs of DDS designed for intra-articular administration^31^ is the homogeneity of size distribution, since it ensures more stable systems^32,33^.

In here, P68 contributed to produce NLC of smaller sizes and monodisperse distribution, as previously described for other NLC formulations^8^, and due to the reduction of the interfacial tension between the lipid phase and water^34^. According to the Experimental Design results, an optimized formulation composed of 20% total lipids (70/30, SL/LL), 2% P68 and 3% NPX was selected for the subsequent tests. This formulation displayed excellent NPX upload capacity, as expected from the NSAID high hydrophobicity^35^. On the other hand, P68 – that represents less than 0.3% of the molecules in a single particle (Table S6), was able to sterically stabilize the nanoparticles for a year at room temperature, without increasing the toxicity of the formulation. Such high stability was also a result of the combination of SL and liquid lipids (LL) that confer a low degree of crystallinity to the NLC core, favoring drug upload^36^. Likewise, the similarity between micrographs obtained with freshly prepared and 12 months-stored samples denotes the stability of the formulation (Fig. 2).

It is worth mentioning the use of an innovative and complementary method for size determination, called NTA. Such number-based video-tracking approach considers the trajectory of individual particles^37^ instead of the average scattered light, as DLS. These different fundamentals explain why average particle sizes determined by NTA can be slightly smaller than those determined by DLS^38^. But NTA also counts the number of particles in a known sample volume, determining the nanoparticles concentration^37^. Based on NTA results, encapsulation of NPX slightly increased the number of nanoparticles in suspension (even that nanoparticles size did not change significantly). Indeed, NPX encapsulation led to the formation of more nanoparticles, in which the lipid proportion was kept the same, but the total number of excipient molecules (SL + LL + surfactant) per particle decreased, in order to accommodate NPX molecules (Table S6). The number of nanoparticles also provided insights on the molecular organization of the NLC particle: each containing *ca*. 10^10^ molecules/particle, with an estimated drug/total lipid (SL + LL) molar ratio of ~1/3 (176.10^5^ NPX molecules to 386.10^5^ SL + 195.10^5^ LL - see Table S6) compatible with NPX upload in the inner lipid core, as expected for this highly hydrophobic molecule^39^.

In the ATR-FTIR analysis, the spectroscopic profile of NLC-NPX sample resembled that of pure NLC, confirming that NPX, despite of its high %EE, did not disrupt the nanoparticles structure, as also confirmed by TEM. These results did not exclude the existence of specific interactions between NLC and NPX available groups (Fig. 3), which probably contribute to the high encapsulation efficiency of the system.

Furthermore, calorimetric measurements revealed thermodynamic features of NLC-NPX formulation. The lower transition observed for the residual CP peak probably results from its interaction with the liquid lipids, decreasing the crystallinity of the nanoparticles core^22,40^. Moreover, the lack of the endothermic transition assigned for NPX in the NLC-NPX sample can be a sign of the solubilization of the NSAID in the nanoparticles (as pictured by the NTA data – Table S6). These results, allied to the high %EE achieved, indicates that NLC is able to protect NPX against degradation, as expected for an efficient DDS. In general, the different techniques used provided a robust *in vitro* characterization dataset, revealing that interaction of NPX with the lipid nanoparticles did not change their structural properties.

The NPX release profile from NLC was best fitted by the Korsmeyer-Peppas model. The results clearly indicate that encapsulation into NLC doubled the time required for delivery of the NSAID. The determined value of the release exponent (*n* = 0.96) - that describes the transport mechanism, indicates a case II transport^24^, in which the drug did not show an initial burst release, but a rather positive exponential non-Fickian release over the time, as the prevailing mechanism of sustained release.

Biological assays were carried out to confirm the safety and efficacy of NLC-NPX optimized formulations. The toxicity profile of NLC is modulated by the diversity and different proportion of the excipients^11^. Here, the cytotoxic effect of the nanoparticles was tested in two different cell lines. NLC (without NPX) at higher concentrations (>10^11^ particles/mL) promoted a decrease in 3T3 and HaCat cells viability, after 24 h of treatment. Similar results have been previously observed in intraperitoneal macrophages treated with SLN composed of CP and P68, in which a decline in cell viability (to less than 50%) was mainly attributed to the surfactant^40^. On the other hand, the protective effect of the nanoparticles against the intrinsic NPX toxicity was evidenced by the significant increase in the viability of both cell lines, after the NPX encapsulation.

In rats, the anti-inflammatory effect of NLC-NPX was demonstrated by the significant decrease in the leucocyte migration to the inflamed TMJ tissue. The effectiveness of the formulation was also confirmed by the attenuation of (TNF-α and IL-1β) inflammatory cytokines levels after treatment with NLC-NPX, for 10 days. This impressive therapeutic effect can be explained by the NPX sustained release profile, prolonging its anti-inflammatory effect for more than one week after application.

The biological performance of the NLC-NPX formulation will certainly reflect in clinical terms, where no multiple administrations will be required, as currently occurs for commercial NSAIDs. The prolonged action can increase the patient compliance to the treatment, while reducing NPX side effects. Based on these encouraging results for NLC-NPX formulation, our further efforts will be directed to evaluate formulations in clinical trials.

## Conclusions

In this work we propose an innovative NLC formulation for the sustained release of NPX aiming the treatment of TMJ disorders. Experimental design (2^3^) selected an optimized formulation based on its physicochemical features. The NLC-NPX showed excellent upload capacity and suitable structural properties, being stable for 12 months of storage at 25 °C. The sustained release of NPX from NLC prolonged its anti-inflammatory effect in the injured TMJ of rats, for more than a week. These achievements point out NLC-NPX as promising treatment of temporomandibular joint and other intra-articular inflammations.

## Materials and Methods

### Materials

NPX base (M.W. 230.259) and P68 (average M.W = 8350) were supplied by Sigma (USA); Dhaymers Quím. Fina (Brazil) provided CP (M.W = 480.862); CCT (M.W = 408.576); was purchased from Lipo Brasil Ltda (Brazil); acetonitrile was supplied from J. T Baker (USA). Deionized water (18 MΩ) was obtained with Elga USF Maxima Ultra-Pure Water equipment.

### Preparation of nanostructured lipid carriers

NLC and NLC-NPX formulations were prepared by the emulsification-sonication method, according to the procedure described by Ribeiro *et al*.^9^ CP (solid lipid, melting point = 53.7 °C)^40^ and CCT (liquid lipid) were heated to 65 °C in water bath, and kept at this temperature throughout the process. In NLC-NPX samples, NPX was solubilized in the lipid phase. Separately, an aqueous solution of P68 was kept in a water bath at the same temperature of the lipid phase. Under high stirring (10,000 rpm in an Ultra-Turrax T18 basic, Germany) the aqueous phase was dropped into the lipid phase, and stirred for 3 min. For the NLC formation, the freshly prepared emulsion was sonicated at 50 W and 20 kHz in a tip sonicator (Vibracell, Sonics & Materials Inc, USA) for 25 min, and then cooled to 25 °C in an ice bath. The samples were stored at room temperature.

### Experimental design

A factorial design with three variables at two levels (2^3^) and a triplicate in the central point was conducted to study the influence of the formulation variables on the NLC critical quality attributes: particle size and polydispersity (aiming at minimizing both). Design was elaborated and results were analyzed using the Design Expert^®^ software, version 9.0.6.2 (Stat-Ease Inc., USA). Table S1 shows the factors and levels used. Analysis of variance (ANOVA, 95% confidence level) was applied to evaluate the results^41^.

### Dynamic light scattering analysis

The particle size, its polydispersity index (PDI) and zeta potential (ZP) values of the nanoparticles were analyzed in a Nano ZS90 ZetaSizer (Malvern Instruments, UK) equipment at 25 °C. The samples were analyzed at different determined times of storage at 25 °C (n = 3).

### Nanoparticle tracking analysis (NTA)

A NTA instrument (LM20 - NanoSight, Amesbury, UK - equipped with a 532 nm laser) was used to quantify the concentration of particles in the NLC formulations. The samples were diluted in ultra-pure water and injected (n = 3) in a sterile sample chamber with syringes at 25 °C.

### Encapsulation efficiency (%EE)

NPX encapsulation efficiency (% EE) of the formulations was determined by the ultrafiltration-centrifugation method, using 30 kDa filters (Millipore) and 4100* g*, for 20 min. The non-encapsulated naproxen fraction was quantified by HPLC in a Varian ProStar (Agilent Technologies, USA) chromatograph, using a C18 column (Alcron Luna^®^) at 25 °C. The mobile phase (1.8 mL min^−1^ flow rate) was composed of acetonitrile:water:acetic acid (50:49:1 v:v). The amount of encapsulated naproxen was determined by subtracting the non-encapsulated (free) fraction from the total amount of naproxen (total NPX) in the sample prior to phase-separation, according to Eq. 1:

1 $$ \% EE=\frac{totalNPX-freeNPX}{totalNPX}\times 100$$

The samples (n = 3) were quantified immediately after preparation and after 12 months to evaluate the nanoparticle stability.

### Transmission electron microscopy (TEM)

To evaluate the morphology of the nanoparticles, samples of NLC and NLC-NPX were analyzed by TEM in a Zeiss LEO 906 microscope, at 80 kV, at day 5 and 12 months after preparation. Sample preparation was performed as previously described^40^.

### Fourier transform infrared spectroscopy (ATR-FTIR)

The absorption spectra of NLC and NLC-NPX lyophilized samples in the infrared region (between 4000 and 500 cm^−1^, with 2 cm^−1^ resolution) were obtained by ATR-FTIR either in a Bruker IFS 66 v/S (Bruker, USA) or in a Perkin Elmer Spectrum 65 instrument (Pike Technologies, USA).

### Differential scanning calorimetry (DSC)

The calorimetric curves were obtained in a DSC 2910 (TA instruments, USA), under argon flow (50 mL.min^−1^), at 10 °C.min^−1^ heating rate, in the 0–250 °C range. Samples of excipients and NLC and NLC-NPX formulations were analyzed.

### Naproxen *in vitro* release study

The *in vitro* release of NPX (30 mg/mL) from NLC was compared to an hydroalcoholic solution of NPX (3%), using Franz-type vertical diffusion cells (0.6 cm^2^ permeation area), and 0.1 μm polycarbonate membranes (Millipore^®^). Samples were added to the donor compartment and 5% Tween solution in phosphate buffered saline (PBS) pH 7.4 was placed in the acceptor compartment^42^. At pre-established intervals, aliquots (0.2 mL) from the acceptor compartment were withdrawn and the volume was immediately replaced with 5% Tween-in-PBS solution. Measurements were carried out in triplicate and analyzed by HPLC. In the mathematical modeling of the release kinetics curves, the KinetDS 3.0 software was used^43^. To describe the release mechanisms, the Korsmeyer-Peppas mathematical model was best fitted, according to the equation below:

2 $$k{t}^{n}=Mt/{M}_{\infty }$$

where: M_∞_ is the amount of drug at the equilibrium time; Mt the amount of drug diffused at time t; k the release constant and *n* the release exponent. To determine the *n* value, just the initial curve fraction (covering < 60% drug-release) was considered.

### Cytotoxicity tests

For the tests, Balb/c 3T3 mouse fibroblasts and HaCat human keratinocytes were treated with Dulbecco’s modified eagle medium (DMEM) supplemented with 10% fetal bovine serum and 1% penicillin-streptomycin. The cells (1 × 10^5^ cells/mL for 3T3 or 7 × 10^4^ cells/mL for HaCat) were maintained at 37 °C and 5% CO_2_ for 24 h. Cell viability was measured by the MTT (3-(4,5-dimethylthiazol-2-yl)-2,5-diphenyltetrazolium bromide) test^44^. The cells were treated with the NLC and NLC-NPX formulations (10^4^–10^13^ nanoparticles/mL) and also with a preparation of NPX diluted in culture medium plus 0.1% of dimethyl sulfoxide (DMSO), at concentrations of 2.48.10^−8^–2.48.10^−2^ M, (same concentration as in NLC-NPX samples), and maintained at 37 °C, 5% CO_2_ for 24 h. After that the medium was replaced with MTT (5 mg/mL, diluted in DMEM) was added, and the samples were kept at 37 °C and 5% CO_2_ for 3 h. After removal of the medium, DMSO (100%) was added, and the plates were stirred for 5 min to dissolve the produced formazan crystals. The plate was read at λ = 570 nm (ELX800, BIO-TEK^®^ Instruments Inc., USA) and the results were expressed as percent viable cells, relatively to the control (cells treated with DMEM, and 0.1% DMSO).

### Effect of NLC-NPX on TMJ inflammation model in rats

All animal experimental procedures were approved by the Committee on Animal Research of the University of Campinas (CEUA/UNICAMP #3827-1) and were in accordance with guidelines by the National Council for Control of Animal Experimentation (CONCEA) and ARRIVE^45^. Male Wistar rats (200–250 g) were briefly anesthetized by inhalation of 3% isoflurane (30 s) and pretreated with an intra-TMJ injection (20 μL) of NCL-NPX (0.6 mg). After 15 min, 4 h, 6 h, 12 h, 24 h, 3 d, 7 d or 10 d animals were treated with intra-TMJ injection (50 μL) of carrageenan (100 μg), a non-neurogenic inflammation agent on TMJ, leading to high levels of leukocytes migration^46^. After 1 hour (the peak for leukocyte migration in this inflammatory model), the animals were euthanized by deeply anesthesia. The articular cavity was washed with 10 μL of PBS + 1 mM ethylenediaminetetraacetic acid (EDTA) for leukocyte migration analysis, and periarticular tissues were removed for evaluation of the levels of pro-inflammatory cytokines^47^. Carrageenan intra-TMJ injection was considered the negative control but the low solubility of NPX prevented its use (e.g. pretreatment followed by carrageenan intra-TMJ injection) as the positive control. In order to check if the action of the NLC-NPX was local instead of systemic, a group of animals was pretreated with contralateral intra-TMJ injection of NLC-NPX (c.t.).

To evaluate the effect of NLC-NPX on carrageenan-induced leukocyte migration, the total number of leukocytes was assessed using a Neubauer chamber (expressed in number of cells × 10^4^/cavity), by diluting the exudate in Turk solution (1:2, v-v)^47^.

Evaluation of the effect of NLC-NPX on carrageenan-elicited protein level of TNF-α and IL-1β was performed by the individual homogenization of the periarticular tissues in 500 μL of Ripa Lysis Buffer (Santa Cruz, Biotechnology, Dallas, Texas, USA), with protease inhibitors, and centrifuged at 4,500 g for 10 min at 4 °C. Protein levels of pro-inflammatory cytokines (TNF-α and IL-1β) from TMJ periarticular tissues were quantified by enzyme-linked immunosorbent assay (ELISA). The TNF-α and IL-1β ELISA kits were purchased from R&D Systems (Minneapolis, MN, USA) and used accordingly to the manufacturer’s protocol.

### Ethical conduct of research

The authors state that they have followed the principles outlined in the Declaration of Helsinki for all animal experimental investigations.

## Supplementary information

Supplementary information

## Data Availability

All data generated or analyzed during this study are included in this manuscript.
