# Supplementary material for: Improved efficacy of naproxen-loaded NLC for temporomandibular joint administration
Source: Sci Rep. 2019 Aug 1;9:11160. doi: 10.1038/s41598-019-47486-w (PMC6673697; doi:10.1038/s41598-019-47486-w)
Supplement: Supplementary file 1 — Supplementary information [file 41598_2019_47486_MOESM1_ESM.docx]

**SUPPLEMENTARY MATERIAL**

**Improved efficacy of naproxen-loaded NLC for temporomandibular joint administration**

Viviane A. Guilherme^a^, Lígia N. M. Ribeiro^a^, Ana C. S. Alcântara^b^, Simone R. Castro^a^, Gustavo H. Rodrigues da Silva^a^, Camila Gonçalves da Silva^a^, Márcia C. Breitkreitz^c^, Juliana Clemente-Napimoga^d,e^, Cristina G. Macedo^d^, Henrique B. Abdalla^d^, Ricardo Bonfante^d^, Cintia M. S. Cereda^a^ and Eneida de Paula^a^

^a^ Department of Biochemistry and Tissue Biology, Institute of Biology, University of Campinas (UNICAMP), Campinas - São Paulo, Brazil.

^b^ Department of Chemistry, Federal University of Maranhão – UFMA, São Luis - Maranhão, Brazil.

^c^ Department of Analytical Chemistry, Institute of Chemistry, UNICAMP, Campinas - São Paulo, Brazil.

^d^ Department of Physiological Science, Piracicaba Dentistry University, UNICAMP, Piracicaba - São Paulo, Brazil.

^e^ São Leopoldo Mandic Faculty/Research Institute, Physiology area, Campinas - São Paulo, Brazil.

**Table S1:** Variables and corresponding levels used in the (2^3^ with central point) experimental design. Total lipids in the formulation (SL + LL) = 20 %.

| Variable | Low Level | Central point | High Level |
| --- | --- | --- | --- |
| SL/LL lipid ratio (%) | 70/30 | 72.5 / 27.5 | 75 / 25 |
| Pluronic® F-68 (%) | 1 | 1.5 | 2 |
| Naproxen (%) | 0 | 1.5 | 3 |

**Table S2:** 2^3^ Experimental design for the NLC formulation selection: variable levels (concentration) and responses. SL = solid lipid. Shaded lines reveal the optimized formulation (see text).

|  | **Variables** | | | **Responses** | |
| --- | --- | --- | --- | --- | --- |
| **Formulation** | **A: SL**  **(%)** | **B: P68**  **(%)** | **C: NPX (%)** | **Size (nm)** | **PDI** |
| **1** | 70.00 | 1.00 | 0.00 | 577.00 | 0.61 |
| **2** | 75.00 | 1.00 | 0.00 | 591.10 | 0.40 |
| **3** | 70.00 | 2.00 | 0.00 | 278.70 | 0.19 |
| **4** | 75.00 | 2.00 | 0.00 | 292.70 | 0.22 |
| **5** | 70.00 | 1.00 | 3.00 | 537.00 | 0.46 |
| **6** | 75.00 | 1.00 | 3.00 | 472.20 | 0.32 |
| **7** | 70.00 | 2.00 | 3.00 | 289.50 | 0.19 |
| **8** | 75.00 | 2.00 | 3.00 | 312.80 | 0.25 |
| **9** | 72.50 | 1.50 | 1.50 | 412.60 | 0.21 |
| **10** | 72.50 | 1.50 | 1.50 | 409.70 | 0.25 |
| **11** | 72.50 | 1.50 | 1.50 | 403.80 | 0.22 |

|  | **Table S3:** p-values for *regression* and *lack of fit*.  **A**   \| **Response** \| **p-value for Model** \| **p-value for Lack of fit** \| \| --- \| --- \| --- \| \| Size \| 0.0005 \| 0.0787 \| \| PDI \| 0.0344 \| 0.0503 \|     **Table S4:** Influence of each experimental variable on the “size” response.   \| **Components** \| **p-value** \| **Significant?** \| **Effect type** \| \| --- \| --- \| --- \| --- \| \| A: SL \| 0.6496 \| No \| - \| \| B: P68 \| 0.0001 \| Yes \| Negative \| \| C: NPX \| 0.0172 \| Yes \| Negative \| \| AB \| 0.0456 \| Yes \| Positive \| \| AC \| 0.0795 \| No \| - \| \| BC \| 0.0057 \| Yes \| Positive \| \| ABC \| 0.0454 \| Yes \| Positive \|     **Table S5:** Influence of each experimental variable on the “polydispersity” response.   \| **Components** \| **p-value** \| **Significant?** \| **Effect type** \| \| --- \| --- \| --- \| --- \| \| A: SL \| 0.2553 \| No \| - \| \| B: P68 \| 0.0056 \| Yes \| Negative \| \| C: NPX \| 0.3685 \| No \| - \| \| AB \| 0.0818 \| No \| - \| \| AC \| 0.072 \| No \| - \| \| BC \| 0.2553 \| No \| - \| \| ABC \| 0.8848 \| No \| - \| |  |  |  |  |
| --- | --- | --- | --- | --- | --- | --- | --- | --- | --- | --- | --- | --- | --- | --- | --- | --- | --- | --- | --- | --- | --- | --- | --- | --- | --- | --- | --- | --- | --- | --- | --- | --- | --- | --- | --- | --- | --- | --- | --- | --- | --- | --- | --- | --- | --- | --- | --- | --- | --- | --- | --- | --- | --- | --- | --- | --- | --- | --- | --- | --- | --- | --- | --- | --- | --- | --- | --- | --- | --- | --- | --- | --- | --- | --- | --- | --- | --- | --- |
|  | **Table S6**: NTA results: average size and nanoparticle concentration (mean ± SD), and estimated number of excipient molecules per NLC, calculated from their molar concentration in the formulation.   \| **Sample** \| **Average** \| **Concentration** \| **Composition (. 10^5^ molecules/NLC)** \| \| \| \| \| \| --- \| --- \| --- \| --- \| --- \| --- \| --- \| --- \| \|  \| **Size**  (nm) \| (particles  per mL) \| solid lipid  **(CP)** \| liquid lipid  **(CCT)** \| surfactant  **(P68)** \| \| Drug  **(NPX)** \| \| **NLC** \| 207.9 ± 11.1 \| 3.0 ± 0.5 x 10^13^ \| 605 \| 305 \| 5 \| - \| \| \| **NLC-NPX** \| 206.9 ± 3.9 \| 4.7 ± 0.4 x 10^13^ \| 386 \| 195 \| 3 \| 176 \| \| |  |  |  |  |

**Table S7**: Balb/c 3T3 cell viability as evaluated by the MTT test after treatment with NPX, NLC or NLC-NPX for 24 h. [NPX] = naproxen concentration in NPX and NLC-NPX samples.

| **Nanoparticles** | | **[NPX]** | | **NLC** | | **NLC-NPX** | | **NPX** | |
| --- | --- | --- | --- | --- | --- | --- | --- | --- | --- |
| **/ mL** | **mM** | | **mg/mL** | **Mean** | **SD** | **Mean** | **SD** | **Mean** | **SD** |
| 1.00E+04 | 2.48E-08 | | 5.71E-09 | 97.0 | 1.2 | 94.7 | 5.7 | 91.9 | 5.1 |
| 1.00E+05 | 2.48E-07 | | 5.71E-08 | 95.1 | 4.7 | 95.3 | 2.7 | 93.2 | 1.1 |
| 1.00E+06 | 2.48E-06 | | 5.71E-07 | 90.5 | 3.9 | 90.4 | 6.3 | 88.2 | 6.5 |
| 1.00E+07 | 2.48E-05 | | 5.71E-06 | 84.5 | 4.3 | 89.4 | 5.4 | 89.3 | 5.5 |
| 1.00E+08 | 2.48E-04 | | 5.71E-05 | 85.6 | 4.5 | 92.8 | 3.5 | 85.4 | 2.7 |
| 1.00E+09 | 2.48E-03 | | 5.71E-04 | 78.2 | 3.8 | 89.2 | 0.5 | 84.6 | 5.3 |
| 1.00E+10 | 2.48E-02 | | 5.71E-03 | 66.3 | 7.1 | 74.1 | 4.9 | 78.2 | 4.1 |
| 1.00E+11 | 0.248 | | 0.0571 | 63.5 | 2.5 | 62.7 | 4.2 | 57.1 | 1.6 |
| 1.00E+12 | 2.48 | | 0.571 | 39.3 | 3.6 | 33.1 | 4.0 | 19.7 | 2.4 |
| 1.00E+13 | 24.8 | | 5.71 | 23.7 | 1.9 | 18.1 | 0.3 | 10.3 | 0.7 |

**
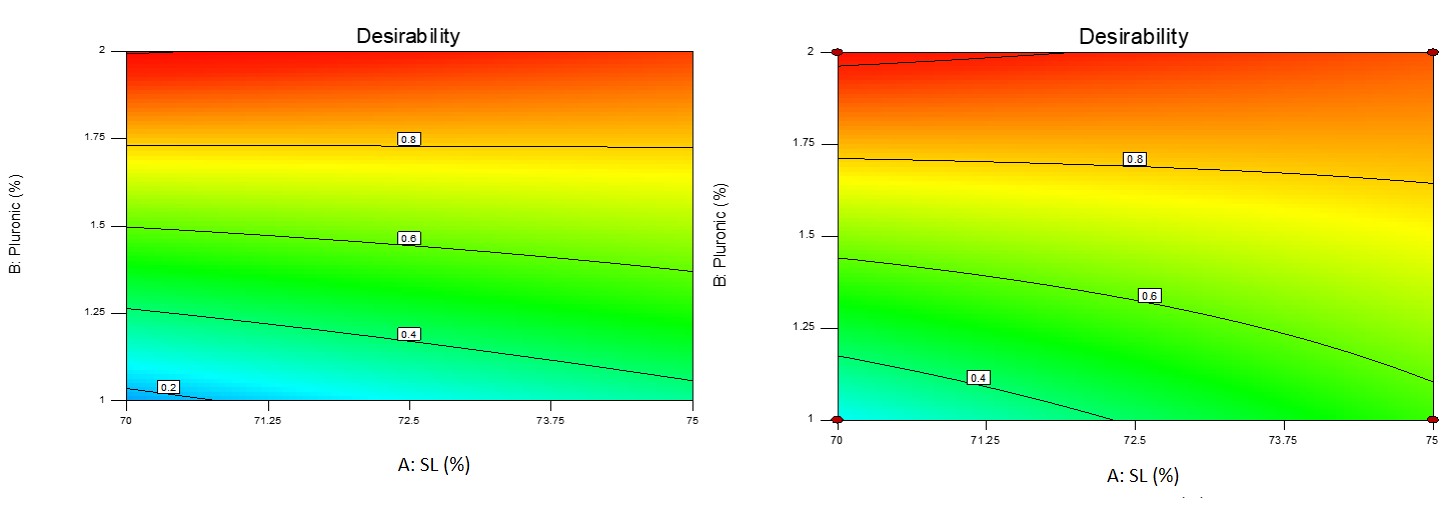
A B**

**Figure S1:** Desirability plots for NLC samples: A) without naproxen and B) with naproxen. The red stripe indicates formulations with smaller size and smaller PDI.
